# Unraveling the phosphorylation profiles of human‐derived Tau species and their role in seeding competency and Alzheimer's disease progression

**DOI:** 10.1002/alz70855_103417

**Published:** 2025-12-23

**Authors:** Mariana Martins, Dhanush Sivasankaran, Noé Quittot, Bradley T. Hyman

**Affiliations:** ^1^ Massachusetts General Hospital, Harvard Medical School, Boston, MA, USA

## Abstract

**Background:**

The aggregation and propagation of the microtubule‐associated protein Tau is a hallmark of Alzheimer's disease (AD) pathogenesis. Aggregated Tau aqueous‐extractable species act as seeds that template the aggregation of normal Tau proteins in a prion‐like manner, and this so‐called seeding competency correlates with AD progression. However, the molecular drivers of the prion‐like aggregation of Tau still need to be understood. Although Tau hyperphosphorylation has been shown to play an important role in its seeding competency, it remains unclear whether some phosphorylation sites are more influential than others. This study aims to determine how Tau's phosphorylation profile affects its seeding propensity.

**Method:**

Tau aqueous‐extractable species were extracted from AD human brain tissue, followed by fractionation into high‐molecular‐weight (HMW) and low‐molecular‐weight (LMW) species through size‐exclusion chromatography. HMW Tau was further fractionated into bioactive (heavily post‐translationally modified and more negatively charged) and non‐bioactive (less post‐translationally modified and less negatively charged) species by anion‐exchange chromatography.

**Result:**

Our study shows that lambda protein phosphatase treatments at varying incubation times partially dephosphorylate these Tau species. The phosphorylation profile is systematically characterized via mass spectrometry and biochemical techniques using multiple specific anti‐Tau phospho‐sites. Additionally, using Förster resonance energy transfer (FRET)‐based biosensor cells, we observe that reducing the phosphorylation level reduces the seeding activity of these modified Tau species.

**Conclusion:**

This work provides insights into the interplay between Tau post‐translational modifications and seeding activity, with potential implications for designing more effective therapeutical strategies that modulate Tau seeding capacity and propagation in AD.

